# Development of the Hedonic Overeating–Questionnaire (HEDO–Q)

**DOI:** 10.3390/nu14091865

**Published:** 2022-04-29

**Authors:** Anja Hilbert, Veronica Witte, Adrian Meule, Elmar Braehler, Soeren Kliem

**Affiliations:** 1 Integrated Research and Treatment Center Adiposity Diseases, Behavioral Medicine Research Unit, Department of Psychosomatic Medicine and Psychotherapy, University of Leipzig Medical Center, 04103 Leipzig, Germany; elmar.braehler@medizin.uni-leipzig.de; 2 Clinic for Cognitive Neurology, University of Leipzig Medical Center, 04103 Leipzig, Germany; witte@cbs.mpg.de; 3 Department of Neurology, Max Planck Institute for Human Cognitive and Brain Sciences, 04103 Leipzig, Germany; 4 Department of Psychiatry and Psychotherapy, University Hospital, LMU Munich, 80336 Munich, Germany; ameule@med.lmu.de; 5 Schoen Clinic Roseneck, 83209 Prien am Chiemsee, Germany; 6 Department of Psychosomatic Medicine and Psychotherapy, University Medical Center of the Johannes Gutenberg University of Mainz, 55131 Mainz, Germany; 7 Ernst-Abbe-Hochschule, University of Applied Sciences, 07745 Jena, Germany; soeren.kliem@eah-jena.de

**Keywords:** food addiction, addictive-like eating, hedonic overeating, eating disorders, obesity, wanting, liking, psychometric, reliability, validity

## Abstract

Addictive-like eating is prevalent, but a clear conceptualization and operationalization outside of an addiction framework is lacking. By adopting a biopsychological framework of food reward, this study sought to develop and evaluate a brief self-report questionnaire for the trait assessment of hedonic overeating and dyscontrol. Items in the Hedonic Overeating–Questionnaire (HEDO–Q) were constructed following a rational approach and psychometrically evaluated in a large random sample from the German population (*N* = 2531). A confirmatory factor analysis supported the unidimensional nature of the six-item HEDO–Q with the three postulated components of wanting, liking, and dyscontrol. Psychometric properties were favorable with good corrected item-total correlations, acceptable item difficulty and homogeneity, and high internal consistency. Population norms were provided. The HEDO–Q revealed strict measurement invariance for sex and partial invariance for age and weight status. Discriminant validity was demonstrated in distinguishing participants with versus without eating disturbances or obesity. Associations with the established measures of eating disorder and general psychopathology supported the convergent and divergent validity of the HEDO–Q. This first evaluation indicates good psychometric properties of the HEDO–Q in the general population. Future validation work is warranted on the HEDO–Q’s stability, sensitivity to change, and predictive and construct validity.

## 1. Introduction

Worldwide, obesity, an excess accumulation of body fat, defined through a body mass index (BMI) ≥ 30.0 kg/m^2^ [1], has more than tripled over the past three decades [2]. This pandemic rise in obesity, especially in Western industrialized countries, is often attributed to excessive energy intake fostered by the obesogenic environment with ubiquitous access to highly palatable and calorific but unhealthy food [3,4]. Citing similarities in neurobiological reward processes between obesity and substance-based addictions, the food addiction (FA) hypothesis [5] postulates that specific foods, particularly those high in sugar or fat or highly or ultra-processed foods, may provoke addictive behavioral responses in vulnerable individuals, characterized by high impulsivity and reward sensitivity, by over-activating reward-related brain circuits, leading to desensitization [6]. Relatedly, the definition of FA follows the criteria for addictive disorders, including substance dependence in the *Diagnostic and Statistical Manual of Mental Disorders Fourth Edition (DSM–IV)* [7], or substance use disorder in the *Fifth Edition* (DSM–5) [8]. Notwithstanding, the validity and clinical utility of a potential clinical FA diagnosis still need to be demonstrated [9].

Since its introduction, the FA hypothesis has been energetically and controversially discussed [10,11,12,13,14]. FA has mostly been operationalized through self-report, using the empirically supported Yale Food Addiction Scale (YFAS 2.0 for the DSM–5) [15] for the trait assessment of addictive behavioral responses towards certain foods, for example, those high in sugar or fat, over the last 12 months. From the 35 items (or 13 items for the abbreviated mYFAS 2.0), a continuous symptom count is calculated by summing the 11 DSM–5 symptoms for substance use disorders [5]. FA is considered to be present if ≥2 symptoms (e.g., strong cravings, unsuccessful attempts to cut down, overeating despite negative consequences, withdrawal, tolerance) and clinically significant impairment/distress are present. The main critique of the FA hypothesis pertains to the self-report assessment when adhering to a clinical addiction framework, as well as the relevance of diagnostic criteria of substance use disorder for eating. For example, certain criteria of substance use disorder such as physical symptoms of withdrawal (e.g., trembling, restlessness, seizures) or tolerance have not been demonstrated regarding food in humans [16]. Most importantly, there is a lack of evidence that specific foods contain biochemical addictive substances [16,17]. In addition, despite similarities between obesity and substance use disorder in neurobiological reactions to food and food stimuli versus drugs, substantial differences have been documented [18], and neuroimaging studies have not equivocally supported a severe or consistent perturbance of functional responses to food stimuli in the main brain regions implicated in reward processing [16,19]. Notwithstanding, the concept of FA is widely appealing not only to researchers, but also to the lay public [20], with self-labeled “food addicts”. For example, those with FA show preferences for highly processed foods high in fat and refined carbohydrates [21], overeating behavior [22], and perceptions of symptoms of withdrawal and tolerance [23]. Implications of the FA concept for treatment, however, still remain unclear [6].

In order to circumvent the much-criticized causal assumption of addictive substances such as foods, it was proposed that eating itself could be addictive and that such an addictive-like eating (ALE) could form the clinical presentation of a behavioral addiction or “eating addiction”. In fact, among the eating disorders, the obesity-associated binge-eating disorder (BED), characterized by recurrent binge eating (i.e., consumption of unambiguously large amounts of food with the subjective feeling of losing control over eating) in the absence of regular weight-compensatory behaviors [8], was proposed to represent a behavioral eating addiction [11,24], although the DSM–5 diagnostic criteria of BED do not follow an addiction framework [8]. While trait FA and BED as a clinical eating disorder share certain behaviors (e.g., strong cravings for food, especially if high in fat or sugar), FA determined through the YFAS 2.0 was found to be present in most but not all women with BED (80%), but it was also present at very high rates in women with bulimia nervosa (approximately 95%) and even in those with anorexia nervosa (71%), particularly co-occurring with the binge-purge subtype [15]. This suggests that the FA trait is prevalent across the eating disorders, especially if binge-eating episodes are present, but that not all individuals with eating disorders or those with BED exhibit this trait. Likewise, although proposed initially for obesity, FA presents across the weight range, with individuals with obesity significantly more frequently affected (19.3%) than those with overweight (10.0%) or normal weight (12.2%) [25]. 

Importantly, there is still no consensus on how to conceptualize and measure ALE [6,10] if not related to FA. Referring to dual-process theories of motivation, highlighting interactions between a hedonic appetitive system and a cognitive control system, Ruddock et al. [26] developed the Addiction-like Eating Behavior Scale with two subscales: Appetitive Drive, mostly including items on overeating, and Low Dietary Control, mostly including items on dietary restraint and violations. While the initial psychometric characteristics were favorable, the scale was criticized for adhering to the substance-based framework, assessing eating problems specifically with high-fat/high-sugar, processed, or unhealthy foods and using items derived from descriptions of self-identified “food addicts” [27], while thought to address ALE in a behavioral addictions perspective without specifically defining an addictive substance [28]. 

A greater understanding of ALE was expected from adopting a broader biopsychological framework of food reward [14] that distinguishes two state-dependent processes: the motivational component of wanting (i.e., incentive salience), commonly defined as a food craving or a strong desire or urge for food, and the hedonic component of liking [29], defined as a qualitative-evaluative experience of pleasure [30]. Both components are based on dissociable, partly overlapping neural pathways [31,32], with wanting being a result of the activation of a large and robust brain system including mesocorticolimbic dopaminergic pathways, while liking results from smaller hedonic hotspots in the limbic system, with the involvement of opioid motivation circuits. Importantly, while both wanting and liking form normative hedonic processes of regular eating, hedonic overeating or ALE could result from an adaptation to the obesogenic food environment [12,14] in terms of an incentive sensitization, magnifying wanting and detaching it from liking [33]. 

Behaviorally, wanting and liking have mostly been measured as state-based through direct assessment via explicit self-report. For example, among multiple experimental paradigms used for measurement [34], the presumably most popular Leeds Food Preference Questionnaire [35], a computerized task, measures explicit wanting and explicit liking though visual analogue scales, in addition to implicit wanting operationalized through a forced choice procedure. The state measures of explicit wanting and explicit liking were—to varying degrees—associated with conceptually similar trait measures. For example, positive associations between explicit liking and trait disinhibition have been found [36,37], whereas associations of explicit wanting and explicit liking with trait measures of binge eating were heterogenous [34,38]. Given that most psychological constructs include both stable, dispositional trait components and fluctuating, situational state components [39], it is notable that food-related wanting and liking have not been operationalized as traits. A number of self-report questionnaires or subscales cover single trait aspects of wanting and liking, for example, craving (e.g., Food Cravings Questionnaires) [40,41] or reward-based eating (e.g., Power of Food Scale [42]; Reward-based Eating Drive 13 [43,44]), most of which are lengthy and do not offer a joint operationalization. 

To the best of our knowledge, a self-report questionnaire for the trait assessment of explicit wanting and explicit liking is lacking. Neuroimaging research suggests that the obesogenic environment favors—in vulnerable persons—a hedonic rather than homeostatic control of food intake, which—coupled with impaired inhibitory control [45] —may increase the likelihood of hedonic overeating and weight gain [46]. Based on this research, we propose to operationalize not only trait wanting and trait liking in a new self-report questionnaire, but also dyscontrol or a breakdown of cognitive control over eating, a component of ALE derived from qualitative research [47]. In addition, dyscontrol, as assessed with cognitive inhibition tasks, was found to be associated with decreased prefrontal activation in individuals with BED, bulimia nervosa, and obesity [48]. From a neuroeconomic perspective, eating decisions, assessed using (implicit) bedding paradigms on food items according to a “willingness to pay” theory, result from integrating diverse food-related signaling, including the motivation, liking, and controlling of adverse eating-related consequences in a subjective value encoded in the orbitofrontal cortex, thereby initiating motor behaviors and eventually (non)action [49]. The predominantly prefrontal cognitive control system [50] is thought to interact with the reward system, providing top-down regulation of food-related attention, decision-making, and motivation [51], which provides further justification for joint assessment. In this context, the goal of this study was to develop and evaluate a trait self-report measure of food-related wanting, liking, and dyscontrol for the study of hedonic overeating in population-based or clinical research in a brief, economic format.

## 2. Materials and Methods

### 2.1. Participants

A random sample of the German population was drawn for a survey investigation on physical and mental well-being in May–July 2019, with assistance from a demographic research institute (USUMA, Berlin, Germany). A reference system for representative studies in Germany was used for sampling (https://www.adm-ev.de/en/services/the-adm-sampling-system accessed on 4 March 2019). Accordingly, a three-stage random sampling procedure served to determine sample point regions based on representative data; target households within regions; and target persons within households.

Pursuing this approach, 5393 noninstitutionalized civilians ≥14 years of age with sufficient German language skills were selected, of which 2542 individuals participated in the survey, corresponding to a response rate of 47.1% (households: 735 not reached, 1233 refused; target persons: 193 not reached, 29 incapacitated, 661 refused). Missing data led to the further exclusion of 11 participants (0.2%), leaving a final sample of *N* = 2531 individuals for this study.

The study was approved by the Ethics Committee of the University of Leipzig (No. 145/19-ek). Trained assessors visited the participants in person, informed them about the study procedures in a verbal and written format, and obtained informed consent. For minor participants, one parent was additionally required to provide informed consent. Participants were first interviewed by the assessors with a structured questionnaire on basic sociodemographic information and then were asked to complete the self-report questionnaires (cf. Section 2.2) on their own, while the assessor remained available to help if needed.

The total study sample consisted of 1350 women (53.3%) and 1181 men (46.7%) with a mean age of 48.4 years (*SD* = 17.9) and a mean BMI of 25.82 kg/m^2^ (*SD* = 5.02), calculated from self-reported height and weight (Table 1). Regarding weight status, 48.2% of participants were classified as normal weight or underweight, 36.5% were classified as overweight, and 14.3% were classified as obese [1].

### 2.2. Measures

#### 2.2.1. Hedonic Overeating–Questionnaire

The development of the Hedonic Overeating–Questionnaire (HEDO–Q) to assess wanting (food craving; anticipatory reward component), liking (pleasure to eat; consummatory reward component), and dyscontrol (loss of control over eating) followed established recommendations for scale development [52,53]. In phase I (item development), an item pool was constructed for the assessment of the three theoretically derived domains (cf. Section 1). Sources for item construction were descriptions of hedonic overeating or ALE of patients with obesity and BED (*N* = 15) at the Obesity Outpatient Unit at Leipzig University Medical Center, and self-report questionnaires targeting similar content were inspected (e.g., Addiction-like Eating Behavior Scale [26], Reward-based Eating Drive 13 [54], Power of Food Scale [42,55], Yale Food Addiction Scale [56], Self-regulation of Eating Behavior Questionnaire [57,58], Eating Inventory [59,60], and Eating Disorder Examination-Questionnaire [61,62]). Note that these questionnaires served as sources of inspiration, but the HEDO–Q does not include any verbatim or adapted items from these questionnaires. The resulting item pool of 52 items was evaluated for content validity, in order to ensure that an item adequately assessed the given domain. Items were required to be applicable across the spectrum of weight and eating disturbances to normalcy and discriminate between presentations. References to specific foods or food properties and addiction labels were omitted. Items pertaining to potential antecedents (e.g., mood, restrained eating, food cues) or consequences of eating (e.g., fear of weight gain) were excluded, which resulted in a reduced item pool of 18 items. In phase II (scale development), these 18 items were reviewed by research psychologists (*N* = 5) with a specialty in eating and weight disorders at Leipzig University Medical Center. Specifically, experts individually rated each item regarding validity and comprehension on a 5-point Likert scale. Subsequently, the 9 items with the highest ratings were selected for the preliminary questionnaire, a feasible length for epidemiological applications. The preliminary HEDO–Q was provided with a 5-point scale (0 = never to 4 = always) containing three items each targeting wanting, liking, and dyscontrol, one of which was reverse-scored. In phase III (scale evaluation, see below), the three reverse-scored items had to be removed because of psychometric deficiencies (cf. Section 2.3). For the final six items of the HEDO–Q, a total mean score and the mean scores of wanting, liking, and dyscontrol were computed, with higher scores indicating greater hedonic overeating.

#### 2.2.2. Eating Disorder Examination–Questionnaire

For convergent and divergent validation, the 8-item short form of the eating Disorder Examination-Questionnaire (EDE–Q) [61,62], the EDE–Q8 [63], was used to assess global eating disorder psychopathology. Two items each cover the concepts of restraint, eating concern, weight concern, and shape concern and are provided with a 7-point Likert scale (0 = characteristic was not present; 6 = characteristic was present every day/in extreme form). A mean global score is calculated, with higher scores indicating greater eating disorder psychopathology. Internal consistency based on this study’s data was excellent (Cronbach’s α = 0.919, 95% CI 0.909–0.928; McDonald’s ω total = 0.937, 95% CI 0.933–0.941). We expected small-sized associations of the EDE–Q8 with noneating-disorder-specific liking and medium-to-large-sized associations with wanting and dyscontrol.

#### 2.2.3. Patient Health Questionnaire

The Patient Health Questionnaire–4 (PHQ–4) [64], the ultra-short form of the 78-item PHQ [65] with 4 items, was used for determining divergent validity and assessing depressive symptoms (PHQ–2) and generalized anxiety disorder symptoms (GAD–2). Rated on a 4-point Likert scale (0 = not at all; 3 = nearly every day), items are summed up to scale scores, with higher scores indicating greater psychopathology. Both the PHQ–2 and GAD–2 had an acceptable-to-good internal consistency (PHQ–4: Cronbach’s α = 0.843, 95% CI 0.827–0.858; McDonald’s ω total = 0.843, 95% CI 0.825–0.861). We assumed small-sized associations with the HEDO–Q.

#### 2.2.4. Measures for Discriminant Validation

Discriminant validity was determined for weight status, comparing participants with obesity (BMI ≥ 30.0 kg/m^2^) versus those without obesity (BMI < 30.0 kg/m^2^; cf. Section 2.1.), and eating disturbances, comparing those with versus without these disturbances. Eating disturbances were operationalized using two EDE–Q items [61,62], assessing the number of objective binge-eating episodes and compensatory behaviors (aggregated assessment of self-induced vomiting, laxative misuse, and driven exercising) over the past 28 days, dichotomized to any objective binge eating and/or any compensatory behaviors. Based on the population-based research regarding self-reported hedonic hunger, general food craving, and binge eating in the population [55,66,67], we expected greater hedonic overeating in subsamples with versus without obesity and eating disturbances, respectively, but no differences by sex.

### 2.3. Data Analytic Plan

We applied chained equation modeling [68] using the variables age, sex, weight status, household income, migration background, and marital status to estimate missing data (<1% for each item of the HEDO–Q). To avoid unrealistic item values, the estimated values (ŷ) were corrected using predictive mean matching (i.e., the realistic values closest to the predicted value were chosen). We used the R package mice [69] for this analysis. To evaluate the factorial validity of the HEDO–Q, a higher order factor model with four three-order factors comprising three postulated subscales was analyzed using confirmatory factor analysis (CFA). (Three negatively polarized items with reversed wording were excluded in an initial data analysis phase due to psychometric deficiencies). We used robust maximum likelihood estimation (MLM) with the mean-adjusted Satorra-Bentler χ^2^ statistic, which has been shown to be robust to the violation of normality. Model fit was assessed using the Comparative Fit Index (CFI), the Tucker Lewis Index (TLI), the Standardized Root Mean Square Residual (SRMR), and the Root Mean Square Error of Approximation (RMSEA). Per convention, CFI and TLI > 0.900 indicate an adequate model fit and CFI and TLI > 0.950 indicate a good fit [70]. SRMR < 0.08 is considered to represent a good fit [70]. RMSEA values <0.050 represent a close fit, values between 0.050–0.080 represent a reasonably close fit, and values >0.080 represent an unacceptable fit [71]. 

Furthermore, we conducted measurement invariance analyses across sex, age, and weight status using the sequential strategy for second-order factorial models described by Chen, Sousa, and West [72,73]. The following steps were subsequently tested: configural invariance (the observed variables are correlated with the same latent constructs in both groups), weak invariance (allowing for the comparison of structural relationships between latent constructs in groups), strong invariance (allowing for the comparison of means of the latent construct between groups), and full invariance (leading to comparable reliabilities of indices in groups and comparable decisions in screening processes).

Based on the second-order model, seven models resulted. As recommended by Chen, CFI differences with a cut-off value of ΔCFI > −0.01, in addition to RMSEA changes >0.015, were used for testing the different stages of measurement invariance. In the case that one or more model parameters differed among groups, the specific parameter was freed, and we performed further invariance tests as long as at least two invariant parameters were found per invariance test (i.e., partial measurement invariance) [74]. Data analyses were carried out using the R package lavaan [75]. 

Regarding construct validity, independent sample *t* tests were calculated for determining discriminant validity on the HEDO–Q item, component, and total score level. Pearson’s *r* correlation coefficients served to determine associations between the HEDO–Q components and total score, respectively, and measures for convergent and divergent validation (EDE–Q8, PHQ–2, GAD–2). Item difficulty was calculated as *P_i_* = *x_i_*/max(*x_i_*) × 100. The Shapiro–Wilk statistic was used to examine the fit of the empirical distribution to the normal curve. The test statistic *W* can be interpreted similar to a correlation coefficient, with values between 0 and 1, similar to the coefficient of determination. The closer the test statistic is to 1, the less deviation the actual variance shows from the hypothetical variance, thus speaking for a normal distribution. Corrected item-total correlations and the average inter-item correlation were calculated using Pearson’s *r*. Internal consistency was determined as Cronbach’s α and McDonald’s ω total with 95% confidence intervals (CI). Internal consistency on the components’ level with two items each was estimated using Spearman–Brown correlation coefficients [76].

Data were analyzed using R version 4.1.2 [77]. A two-tailed α < 0.05 was applied for all statistical tests.

## 3. Results

### 3.1. Factor Structure

**Figure 1:**
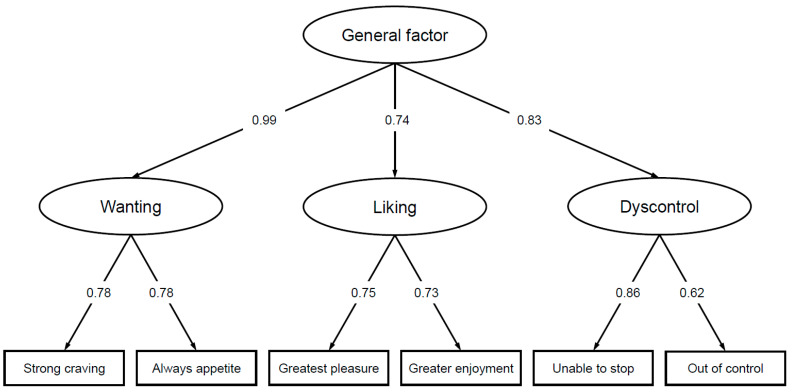
Confirmatory factor analysis of the 6-item Hedonic Overeating–Questionnaire (*N* = 2531): standardized factor loadings for the first- and second-order factors.

The results of the CFA revealed good fit parameters for the second-order factor model (RMSEA = 0.055 [95% CI 0.039–0.072]; SRMR = 0.019; TLI = 0.978, CFI = 0.991). First- and second-order factor loadings are shown in Figure 1.

### 3.2. Item Characteristics

Means and standard deviations and item characteristics of the HEDO–Q are summarized in Table 2. The total mean score was 1.22 (*SD* = 0.71, range 0.00–4.00). The Shapiro–Wilk value, *W* = 0.97, for the HEDO-Q total score was indicative of approximate normality. The corrected item-total correlations for the HEDO–Q total score were good (*r_it_* = 0.61–0.79) [78], the item difficulty ranged between 11.00 (“Out of control”) and 43.50 (“Greatest pleasure”), and the item homogeneity amounted to *r_ii_* = 0.44.

### 3.3. Internal Consistency

The internal consistency of the HEDO–Q total score was high (Cronbach’s α = 0.822, 95% CI 0.808–0.835; McDonald’s ω total = 0.830, 95% CI 0.813–0.836). The Spearman–Brown coefficients for the two-item consistency estimation ranged from *r_sb_* = 0.70–0.75 for the components of wanting, liking, and dyscontrol.

### 3.4. Measurement Invariance

Measurement invariance analyses of the second-order model confirmed (partial) strict invariance regarding sex, age, and weight status. A detailed description of the results can be found in Table 3. 

### 3.5. Norms

Sex, age, and weight status-specific norms of the HEDO–Q are provided in Table 4.

### 3.6. Construct Validity

As hypothesized, all HEDO–Q items, components, and the total score were higher in participants with versus without obesity and in those with versus without an eating disturbance (medium-to-large effect sizes), while variations by sex were not detected (*d* ≤ 0.10). The results for discriminant validity are presented in Table 5.

Regarding convergent and divergent validity, as presented in Table 6, the HEDO–Q total score and the components wanting and dyscontrol showed medium-to-large-size associations with the global eating disorder psychopathology, while liking showed small-size associations, as expected. Associations of the HEDO–Q total score and components wanting and dyscontrol with symptoms of depression and generalized anxiety disorder were small, as hypothesized, and close to zero for liking.

## 4. Discussion

This study provides the first psychometric evaluation of the newly developed HEDO–Q for the assessment of key components of hedonic overeating. In phase I (item development), an item pool of 52 items was constructed, of which 18 items were selected after evaluating content validity. In phase II (scale development), the item pool was further reduced to 9 items based on expert ratings regarding comprehension and validity. In phase III (scale evaluation), the preliminary HEDO–Q was evaluated in a community sample randomly drawn from the German population to be representative for age and sex (*N* = 2531). A CFA showed a unidimensional second-order structure, while reproducing the three postulated first-order components of wanting, liking, and dyscontrol of the 6-item HEDO–Q, after removing three reverse-scored items. Psychometric properties were favorable with a low number of missing values (0.2%); good corrected item-total correlations; moderate-to-high item difficulty; and adequate item homogeneity. The high item difficulty of the dyscontrol items was related to low endorsement, especially of the item “Out of control”, which is in line with epidemiological research on binge eating that was self-reported by 4.2% of respondents in the German population [67]. Internal consistency of the total score was high, as evidenced by Cronbach’s α = 0.822 and McDonald’s ω total = 0.830. The components of wanting, liking, and dyscontrol also were internally consistent, considering the Spearman–Brown coefficients (cf. Table 2). Because of the brevity of the HEDO–Q, the total score is recommended for use in future applications. However, a meaningful use of the components’ scores could be justified in the case of acceptable sample-specific psychometric properties, especially internal consistencies of the two items within a component. 

Population norms specific for sex, age, and weight status were provided. The HEDO–Q revealed strict measurement invariance for sex, indicating a fully equivalent measurement of hedonic overeating across men and women. Partial invariance for age and weight status was given; measurement equivalence was first-order strong across ages and first- and second-order strict across weight status when the intercept or residual variance of the item “Out of control” were freed, respectively. Thus, some caution should be taken in making HEDO–Q comparisons across ages and weight status. Indeed, consistent with this partial measurement invariance for age and weight status, binge eating was found to be more prevalent in younger than older age groups and in those with higher than lower body weight in population-based research [67,79].

The HEDO–Q (as well as its items and components) presented good discriminant validity for weight status and eating disturbance, as hypothesized, with higher scores in those with versus without obesity or eating disturbances, respectively, thus indicating high criterion validity and suggesting potential utility in clinical applications. Also consistent with expectations, variations by sex were not observed [55,66,67]. Future studies may address the HEDO–Q’s sensitivity to discriminate between individuals with objectively determined obesity or diagnosis of an eating disorder based on clinical interview. Given the epidemiological nature of this study, we operationalized eating disturbances through self-report using single items on binge eating and compensatory behaviors from the established EDE–Q [61,62]. 

Regarding convergent validity, the medium-to-large associations between the HEDO–Q and its components of wanting and dyscontrol and global eating disorder psychopathology, measured through the eight-item short form of the EDE–Q [63], indicated similarities of hedonic overeating with eating disturbances, including their tendency to experience strong cravings and to lose control over eating. However, the magnitude of these associations also showed that hedonic overeating is not the same as eating disorder psychopathology, as previously demonstrated for other scales measuring ALE [80]. As expected, liking, the consummatory reward component, which may be more characteristic of some eating disturbances or disorders (e.g., BED) than others (e.g., anorexia nervosa) [81], showed small-size associations with eating disorder psychopathology. Divergent validity was demonstrated through small-size associations of the HEDO–Q and its components wanting and dyscontrol with depressive symptoms and generalized anxiety disorder symptoms. Plausibly, associations of liking and depressive and generalized anxiety disorder symptoms were close to zero; indeed, both of these disorders are characterized by anhedonia [82]. The differences in convergent and divergent validity of wanting versus liking are consistent with neurobiological and experimental research, supporting a distinction between these two concepts [33]. Overall, the HEDO–Q demonstrated good construct validity in this initial psychometric evaluation. 

Regarding strengths and limitations, a large random sample was drawn from the population, aimed to be representative for the sex and age of the German population. The response rate of 47.1% is similar to that of other comparable population-based surveys [83]. Of note, our survey included one measure of sex or gender, which are difficult to be distinguished in German language. Diverse gender was not assessed. Weight and height were measured by self-report, which often involves an underestimation of weight and overestimation of height [84,85]. Indeed, the prevalence of obesity in this study was lower than in population-based studies using objective measures (14.3 vs. 23.6%) [86]. While the distribution of the lower net household income groups roughly corresponded to that in the German population, our sample included a higher proportion of individuals with an income of 2000 –< 3500 EUR and a lower proportion of individuals with an income ≥3500 EUR [87]. Further, individuals with other than German nationality were underrepresented when compared to the respective proportion in the German population (3.6 vs. 12.4%) [88], likely related to various barriers to participation, for example, the inclusion criterion of sufficient German language skills. Future validations of the HEDO–Q in ethnically diverse populations are warranted. A further strength of this study was the use of established measures for convergent (EDE–Q8) [63] and divergent (PHQ–4) [64] validation. Future research is warranted on the convergent validation of the HEDO–Q against conceptually similar trait measures of ALE. Further, it is important to note that wanting and liking can occur independent of a conscious, cognitive representation [31,89]. For example, processes of the gut–brain reward pathways have been found to bypass conscious flavor or aroma sensory perception [89] and, thus, direct self-report assessment of explicit wanting and explicit liking. Deserving of clarification is whether or not indirect experimental assessments that commonly deduce implicit responses from participants’ performance reflect these aspects with more validity [90]. Finally, it will be important to disentangle wanting from liking in their relative contribution to eating behavior or weight management [91,92].

This first evaluation of the new HEDO–Q established good psychometric properties in the population and suggests the utility of this scale to identify hedonic overeating in individuals with obesity and eating disturbances. As the cross-sectional design impedes inferences about a causal relationship between HEDO–Q scores, obesity, and eating disturbances, longitudinal research is warranted to examine predictive validity, in addition to test-retest validity informing about temporal stability. Application of the HEDO–Q in intervention studies could elucidate its sensitivity to change [93]. Initial research suggests that state measures of explicit liking and explicit wanting, as assessed with the Leeds Food Preference Questionnaire [35], differentially respond to behavioral weight loss treatment, with potential relevance for weight loss and maintenance [94]. Whether this applies to state measures only or also to the traits wanting and liking requires further research in clinical studies on behavioral weight loss or maintenance treatment as well as eating disorder treatment. Convergent validation is not only required regarding conceptually similar scales. For example, validation against state and experimental measures under laboratory or free-living conditions, and in conjunction with psycho- or neurobiological assessments, could help to disentangle the contribution of dispositional trait and situational state hedonic overeating to food intake and related processes (e.g., attentional bias, food cue reactivity, or reward system activation [95,96,97]). The brevity of the six-item HEDO–Q, developed and evaluated in German—and translated into English based on a backtranslation procedure with a licensed translator—allows for ease of use in a variety of research and clinical settings. The items, instructions, and full scoring information of the HEDO–Q will be made available for free download on the publication server of Leipzig University (cf. https://behavioralmedicine.net/?page_id=277). Ultimately, assessment of hedonic overeating may further the understanding of this construct and inform the development of novel interventions targeting high levels of this overeating in individuals at risk for—or with fully developed—eating and weight disorders.

## Figures and Tables

**Table 1:** Demographic characteristics of the study sample.

	Total	Women	Men
	(*N* = 2531)	(*N* = 1350)	(*N* = 1181)
Age (years)			
Mean (*SD*)	48.4 (17.9)	49.1 (17.8)	47.7 (17.9)
Median [min, max]	50.0 [14.0, 95.0]	50.0 [14.0, 90.0]	49.0 [14.0, 95.0]
Missing	0 (0%)	0 (0%)	0 (0%)
Body mass index (kg/m^2^)	25.82 (5.02)	25.56 (5.40)	26.12 (4.53)
Missing	23 (1.1%)	18 (1.3%)	5 (0.4%)
Weight status			
Underweight (<18.5 kg/m^2^)	49 (1.9%)	37 (2.7%)	12 (1.0%)
Normal weight (18.5–24.9 kg/m^2^)	1173 (46.3%)	684 (50.7%)	489 (41.4%)
Overweight (25.0–29.9 kg/m^2^)	924 (36.5%)	393 (29.1%)	531 (45.0%)
Obesity class I (30.0–34.9 kg/m^2^)	239 (9.4%)	144 (10.7%)	95 (8.0%)
Obesity class II (35.0–39.9 kg/m^2^)	73 (2.9%)	44 (3.3%)	29 (2.5%)
Obesity class III (≥40.0 kg/m^2^)	50 (2.0%)	30 (2.2%)	20 (1.7%)
Missing	23 (0.9%)	18 (1.3%)	5 (0.4%)
Household income (net, per month)			
500 –< 1000 EUR	176 (7.0%)	95 (7.0%)	81 (6.9%)
1000 –< 2000 EUR	716 (28.3%)	456 (33.8%)	260 (22.0%)
2000 –< 3500 EUR	1042 (41.2%)	491 (36.4%)	551 (46.7%)
≥3500 EUR	544 (21.5%)	276 (20.4%)	268 (22.7%)
Missing	53 (2.1%)	32 (2.4%)	21 (1.8%)
Marital status			
Married/living together	1101 (43.5%)	553 (41.0%)	548 (46.4%)
Married/separated	70 (2.8%)	43 (3.2%)	27 (2.3%)
Single	760 (30.0%)	355 (26.3%)	405 (34.3%)
Divorced	368 (14.5%)	226 (16.7%)	142 (12.0%)
Widowed	220 (8.7%)	167 (12.4%)	53 (4.5%)
Missing	12 (0.5%)	6 (0.4%)	6 (0.5%)
Nationality			
German	2437 (96.3%)	1299 (96.2%)	1138 (96.4%)
Other	90 (3.6%)	47 (3.5%)	43 (3.6%)
Missing	4 (0.2%)	4 (0.3%)	0 (0%)

Notes. Displayed are means (*SD*), median [min, max], and *N* (%) derived from raw data.

**Table 2:** Descriptions and item characteristics of the Hedonic Overeating–Questionnaire (HEDO–Q) (*N* = 2531).

	*M*	*SD*	*r_it_*	*P_i_*	*r_sb_*	*W*
**Wanting**	1.38	0.88		34.50	0.75	0.95
* Strong craving*	1.48	0.94	0.78	37.00		0.89
* Always appetite*	1.29	1.02	0.75	32.25		0.88
**Liking**	1.66	0.94		41.50	0.71	0.96
* Greatest pleasure*	1.74	1.05	0.71	43.50		0.90
* Greater enjoyment*	1.58	1.10	0.79	39.50		0.91
**Dyscontrol**	0.62	0.76		15.50	0.70	0.79
* Unable to stop*	0.80	0.91	0.71	20.00		0.79
* Out of control*	0.44	0.79	0.61	11.00		0.59
**HEDO–Q**	1.22	0.71		30.50		0.97

Notes. Displayed are means and SDs of the HEDO–Q items, components, and total scale (0–4), corrected item-total correlations (*r_it_*), item difficulties (*P_i_*), and Spearman–Brown coefficients for two-item consistency estimation per component (*r_sb_*); *W* = Shapiro–Wilk *W* test of normality.

**Table 3:** Measurement invariance analyses of the Hedonic Overeating–Questionnaire (HEDO–Q) for sex, age, and weight status (*N* = 2531).

	χ^2^	*df*	CFI	ΔCFI	RMSEA	ΔRMSEA	Measurement Invariance
**Sex (female, male)**							
1	51.65	12	0.989		0.060		
2	57.21	15	0.989	0.000	0.055	−0.005	√
3	59.52	17	0.989	0.000	0.051	−0.004	√
4	63.57	20	0.989	0.000	0.047	−0.004	√
5	68.10	22	0.989	0.000	0.045	−0.002	√
6	71.04	25	0.989	0.000	0.042	−0.003	√
7	69.91	31	0.990	+0.001	0.036	−0.006	√
**Age (14–34 years, 35–54 years, 55–98 years)**							
1	47.20	18	0.992		0.051		
2	54.34	24	0.992	0.000	0.044	−0.007	√
3	63.08	28	0.991	−0.001	0.044	0.000	√
4	439.12	35	0.901	−0.090	0.130	+0.086	x
4 part ^1^	68.74	32	0.991	0.000	0.041	−0.003	√
5	73.39	36	0.991	0.000	0.039	−0.002	√
6	80.23	41	0.991	0.000	0.037	−0.002	√
7	115.46	53	0.984	−0.007	0.043	+0.006	√
**Weight Status (nonobese, obese)**							
1	50.13	12	0.989		0.059		
2	75.79	15	0.983	−0.006	0.065	+0.006	√
3	99.34	17	0.977	−0.006	0.071	+0.006	√
4	130.44	20	0.971	−0.006	0.074	+0.003	√
5	148.74	22	0.968	−0.003	0.074	+0.000	√
6	176.41	25	0.963	−0.005	0.075	+0.001	√
7	237.22	31	0.932	−0.031	0.091	+0.016	x
7 part ^2^	179.76	30	0.959	−0.004	0.072	−0.003	√

Notes. Model 1: unrestricted baseline model (configural); 2: invariance of first-order factor loadings (first-order weak); 3: additionally, invariance of second-order factor loadings (first- and second-order weak); 4: additionally, invariance of intercepts of measured variables (first-order strong); 5: additionally, invariance of intercepts of first-order factors (first- and second-order strong); 6: additionally, disturbances of first-order factors constrained across groups (first-order strict); 7: additionally, invariance of residual variances of the measured variables (first- and second-order strict); all fit statistics are robust. CFI = Comparative Fit Index; ΔCFI = CFI differences for the different measurement invariance levels; RMSEA = Root Mean Square Error of Approximation; ΔRMSEA = RMSEA differences for the different measurement invariance levels. ΔCFI≤–0.010 complemented by ΔRMSEA *≥* 0.015 indices a violation of measurement invariance. √ marks measurement invariance for the respective level. ^1^ Freed intercept of item “Out of control;” ^2^ freed residual variance of item “Out of control”.

**Table 4:** Norms of the Hedonic Overeating–Questionnaire (HEDO–Q) in the total sample and in subsamples by sex, age, and weight status.

	Total	Sex		Age			Weight Status	
HEDO–Q Score	Sample *N* = 2531	Women *N* = 1350	Men *N* = 1181	14–34 y *N* = 658	35–54 y *N* = 872	55–98 y *N* = 1001	Nonobese *N* = 2144	Obese *N* = 369
0.00	5	5	4	3	4	6	5	2
0.17	8	9	8	6	8	10	9	3
0.33	13	14	12	10	12	16	14	5
0.50	18	19	17	15	16	23	20	7
0.67	26	27	24	20	23	32	28	12
0.83	35	37	33	28	32	42	38	17
1.00	45	47	43	38	43	52	49	25
1.17	56	57	54	47	53	63	59	32
1.33	65	66	64	56	65	71	69	39
1.5	72	73	72	64	72	78	76	47
1.67	79	80	78	73	78	84	83	54
1.83	85	85	84	79	84	89	89	62
2.00	89	89	89	84	90	92	93	69
2.17	92	91	92	87	92	95	95	73
2.33	94	93	95	91	94	96	97	78
2.50	96	95	96	94	95	97	98	82
2.67	97	97	98	96	97	98	99	88
2.83	98	98	98	98	98	99	99	92
3.00	99	99	99	99	99	99	99	96
3.17	99	99	99	99	99	>99	99	97
3.33	99	99	99	99	99	>99	>99	98
3.50	>99	99	>99	99	99	>99	>99	98
3.67	>99	>99	>99	99	>99	>99	>99	99
3.83	>99	>99	>99	>99	>99	>99	>99	99
4.00	>99	>99	>99	>99	>99	>99	>99	>99

Notes. Norms are presented as HEDO–Q total mean scores and corresponding percentiles. The sample sizes (*N*) of the respective groups are based on the imputed data set (cf. Section 2.3), resulting in deviations from the sample description in Table 1 based on raw data. Percentiles are shown for the total sample, added by sex, age and weight status-specific percentiles. The light gray indicates elevated hedonic overeating (90th–94th percentile), the medium gray indicates high hedonic overeating (95th–98th percentile), and the dark grey indicates very high hedonic overeating (≥99th percentile).

**Table 5:** Group differences by sex, weight status, and eating disturbance using the Hedonic Overeating–Questionnaire (HEDO–Q).

	**Women** **(*N* = 1350)**		**Men** **(*N* = 1181)**		**Group Differences**				
	***M***	***SD***	***M***	***SD***	***t*** **(2529)**	***p***	***d***	**95% CI**	
**Wanting**	1.35	0.88	1.41	0.87	1.69	0.092	0.07	−0.01	0.15
* Strong craving*	1.44	0.94	1.51	0.95	1.81	0.071	0.07	−0.01	0.15
* Always appetite*	1.27	1.03	1.31	1.00	1.23	0.220	0.05	−0.03	0.13
**Liking**	1.63	0.96	1.69	0.93	1.49	0.137	0.06	−0.02	0.14
* Greatest pleasure*	1.71	1.06	1.78	1.03	1.86	0.063	0.07	0.00	0.15
* Greater enjoyment*	1.56	1.11	1.60	1.08	0.79	0.428	0.03	−0.05	0.11
**Dyscontrol**	0.62	0.77	0.61	0.74	−0.27	0.786	−0.01	−0.09	0.07
* Unable to stop*	0.80	0.92	0.80	0.90	0.15	0.880	0.01	−0.07	0.08
* Out of control*	0.45	0.83	0.42	0.79	−0.67	0.502	−0.03	−0.10	0.05
**HEDO–Q**	1.2	0.72	1.24	0.69	1.26	0.209	0.05	−0.03	0.13
	**Nonobese** **(*N* = 2144)**		**Obese** **(*N* = 369)**		**Group Differences**				
	***M***	***SD***	***M***	***SD***	***t*** **(2511)**	***p***	***d***	**95% CI**	
**Wanting**	1.29	0.83	1.92	0.96	13.23	<0.001	0.75	0.86	0.63
* Strong craving*	1.38	0.90	2.05	1.00	12.99	<0.001	0.73	0.84	0.62
* Always appetite*	1.20	0.98	1.80	1.12	10.59	<0.001	0.60	0.71	0.49
**Liking**	1.60	0.94	2.03	0.92	8.17	<0.001	0.46	0.57	0.35
* Greatest pleasure*	1.68	1.04	2.11	1.03	7.40	<0.001	0.42	0.53	0.31
* Greater enjoyment*	1.52	1.08	1.94	1.13	6.98	<0.001	0.39	0.50	0.28
**Dyscontrol**	0.52	0.65	1.18	1.03	16.20	<0.001	0.91	1.03	0.80
* Unable to stop*	0.70	0.83	1.37	1.12	13.37	<0.001	0.75	0.87	0.64
* Out of control*	0.34	0.69	0.99	1.17	14.82	<0.001	0.84	0.95	0.72
**HEDO–Q**	1.14	0.65	1.71	0.83	14.91	<0.001	0.84	0.95	0.73
	**No Eating Disturbance** **(*N* = 1856)**		**Eating Disturbance** **(*N* = 675)**		**Group Differences**				
	***M***	***SD***	***M***	***SD***	***t*** **(2511)**	***p***	***d***	**95% CI**	
**Wanting**	1.18	0.79	1.93	0.88	20.33	<0.001	0.91	0.82	1.01
* Strong craving*	1.28	0.87	2.02	0.92	18.68	<0.001	0.84	0.75	0.93
* Always appetite*	1.09	0.93	1.83	1.04	17.10	<0.001	0.77	0.68	0.86
**Liking**	1.51	0.92	2.07	0.89	13.67	<0.001	0.61	0.52	0.70
* Greatest pleasure*	1.60	1.01	2.13	1.05	11.48	<0.001	0.52	0.43	0.61
* Greater enjoyment*	1.42	1.07	2.01	1.04	12.38	<0.001	0.56	0.47	0.65
**Dyscontrol**	0.42	0.58	1.16	0.9	24.55	<0.001	1.10	1.01	1.20
* Unable to stop*	0.57	0.76	1.41	1.00	22.3	<0.001	1.00	0.91	1.09
* Out of control*	0.26	0.62	0.92	1.05	19.43	<0.001	0.87	0.78	0.96
**HEDO–Q**	1.04	0.61	1.72	0.73	23.68	<0.001	1.06	0.97	1.16

Notes. The sample sizes (*N*) of the respective groups are based on the imputed data set (cf. Section 2.3), resulting in deviations from the sample description in Table 1 based on raw data.

**Table 6:** Associations of the Hedonic Overeating–Questionnaire (HEDO–Q) with depression and generalized anxiety disorder symptoms and eating disorder psychopathology (*N* = 2531).

Variable	1	2	3	4	5	6
**1. HEDO–Q**						
**2. Wanting**	0.88 **					
	[0.87, 0.89]					
**3. Liking**	0.81 **	0.56 **				
	[0.80, 0.82]	[0.53, 0.59]				
**4. Dyscontrol**	0.78 **	0.63 **	0.38 **			
	[0.77, 0.80]	[0.60, 0.65]	[0.35, 0.42]			
**5. Depressive Symptoms**	0.15 **	0.13 **	0.05 *	0.21 **		
**(PHQ–2)**	[0.11, 0.19]	[0.09, 0.16]	[0.01, 0.09]	[0.17, 0.25]		
**6. Generalized Anxiety**	0.16 **	0.15 **	0.04 *	0.23 **	0.73 **	
**Disorder Symptoms (GAD–2)**	[0.12, 0.20]	[0.11, 0.18]	[0.00, 0.08]	[0.19, 0.27]	[0.71, 0.75]	
**7. Global Eating Disorder**	0.41 **	0.37 **	0.21 **	0.46 **	0.24 **	0.26 **
**Psychopathology (EDE–Q8)**	[0.37, 0.44]	[0.34, 0.40]	[0.17, 0.24]	[0.43, 0.49]	[0.20, 0.27]	[0.23, 0.30]

Pearson correlation coefficients are displayed. Values in square brackets indicate the 95% confidence interval. * *p* < 0.05, ** *p* < 0.01.

## Data Availability

Research data are not shared.
